# Combining Novel Thrombectomy Devices for Intracardiac Mass Extraction: The Kong and Godzilla of Mass Extraction

**DOI:** 10.14797/mdcvj.1376

**Published:** 2024-06-03

**Authors:** Hussam Al Hennawi, Ahmad Abulshamat, Ali Sheikh, Hisham Qandeel, Kiril Zakharov, Appa Bandi, Mohammed Qintar

**Affiliations:** 1 Jefferson Abington Hospital, Abington, Pennsylvania, US; 2 Jordan University Hospital, Amman, Jordan; 3 Sparrow Hospital, Lansing, Michigan, US; 4 Michigan State University, Lansing, Michigan, US

**Keywords:** intracardiac mass, transcatheter thrombectomy, AngioVac, Inari FlowTriever

## Abstract

Transcatheter extraction of an intracardiac mass is a newer approach that may lead to nonsurgical treatment of complex cardiac masses. We present a case in which thrombectomy devices were combined to extract a right atrial mass, which highlights new frontiers in the treatment of complex transcatheter mass extraction. The combined use of two transcatheter thrombectomy devices (Kong and Godzilla) may provide a powerful addition to the existing armamentarium.

## Introduction

Surgical extraction remains the standard of care for large intracardiac masses. Transcatheter vacuum-assisted mass extraction has emerged as an effective and safe alternative to open surgical thrombectomy, especially in high-risk patients with right-sided thrombosis or vegetation.^1^ The AngioVac system (AngioDynamics) is approved by the US Food and Drug Administration (FDA) and capable of removing intravascular thrombi or vegetations without the need for thrombolytics. Although the AngioVac device was originally designed and approved for removal of right-sided fresh thrombus, its use has expanded for retrieval of a variety of intravascular materials such as right-sided infective endocarditis vegetations, tumor thrombus, catheter- or device-related thrombus, and intravascular or intracardiac masses.^2,3^ In addition, the Inari FlowTriever system (Inari Medical) has been approved by the FDA for the removal of transient clots in the right atrium. It also is indicated for patients with venous thromboembolism disease, for which a case series was published.^4^ Combining these two devices to remove extremely large and unusual right atrial masses has not been described thus far. Herein, we report a very unusual large right atrial mass extraction using two combined thrombectomy systems in a novel way.

## Case Report

A 60-year-old female who experienced sudden cardiac arrest was resuscitated and was found via chest computed tomography (CT) to have a large pulmonary embolism, for which she received systemic lytic therapy. Echocardiography showed a large mobile mass measuring 3.2 × 2.1 cm in the right atrium attached to the interatrial septum (Video 1). She underwent a sternotomy for right atrial mass excision and reconstruction of her atrial septum with an autologous pericardial patch. Pathology showed the mass was a papillary fibroelastoma with a large thrombus. The patient did well postoperatively and was discharged home on 6 months of rivaroxaban and aspirin.

**Video 1:** During 2-dimensional transesophageal echocardiography (TEE), a mass was observed in the right atrium and later identified as a large mass measuring 7 × 3.0 cm, with the body measuring 3.5 × 3.0 cm and the stalk measuring 3.5 × 1.0 cm; it originated in the superior vena cava and occupied significant space in the right atrium. Intraoperatively, attempts were made to extract the mass using the AngioVac cannula, but its size and chronicity posed challenges despite multiple high-flow suction attempts. Subsequently, the FlowTriever 2 was utilized alongside active suction from the AngioVac to break up the mass while efforts were made to extract it. A mesh disc was employed to prevent embolization during the procedure. Although the main body of the mass was successfully extracted, a sizable peduncle/stalk remained attached to the superior vena cava, necessitating the use of a snare to cut the base of the mass. Ultimately, the mass, along with its stalk, was completely extracted through the AngioVac, resulting in the resolution of the mass with no residual fragments remaining; see also at https://youtu.be/0fA2d-mKOK0.

Six months after her initial presentation, the patient returned to the hospital with lightheadedness and shortness of breath. Repeat echocardiography showed a very large right atrial mass with a large stalk originating from the superior vena cava. The mass was confirmed on CT and transesophageal echocardiography TEE (Video 1), and likely represented a thrombus rather than a return of her papillary fibroelastoma.

A heart team was consulted, and the decision was made to remove the mass using a transcatheter approach, largely to avoid a high-risk redo sternotomy. Since the mass occupied almost 60% to 70% of the right atrium, was likely to be chronic, and originated from the superior vena cava, we planned for dual venous access through the right internal jugular and femoral vein, and we planned to use two novel devices (the AngioVac and Inari FlowTriever) to extract this large mass.

**Figure 1:**
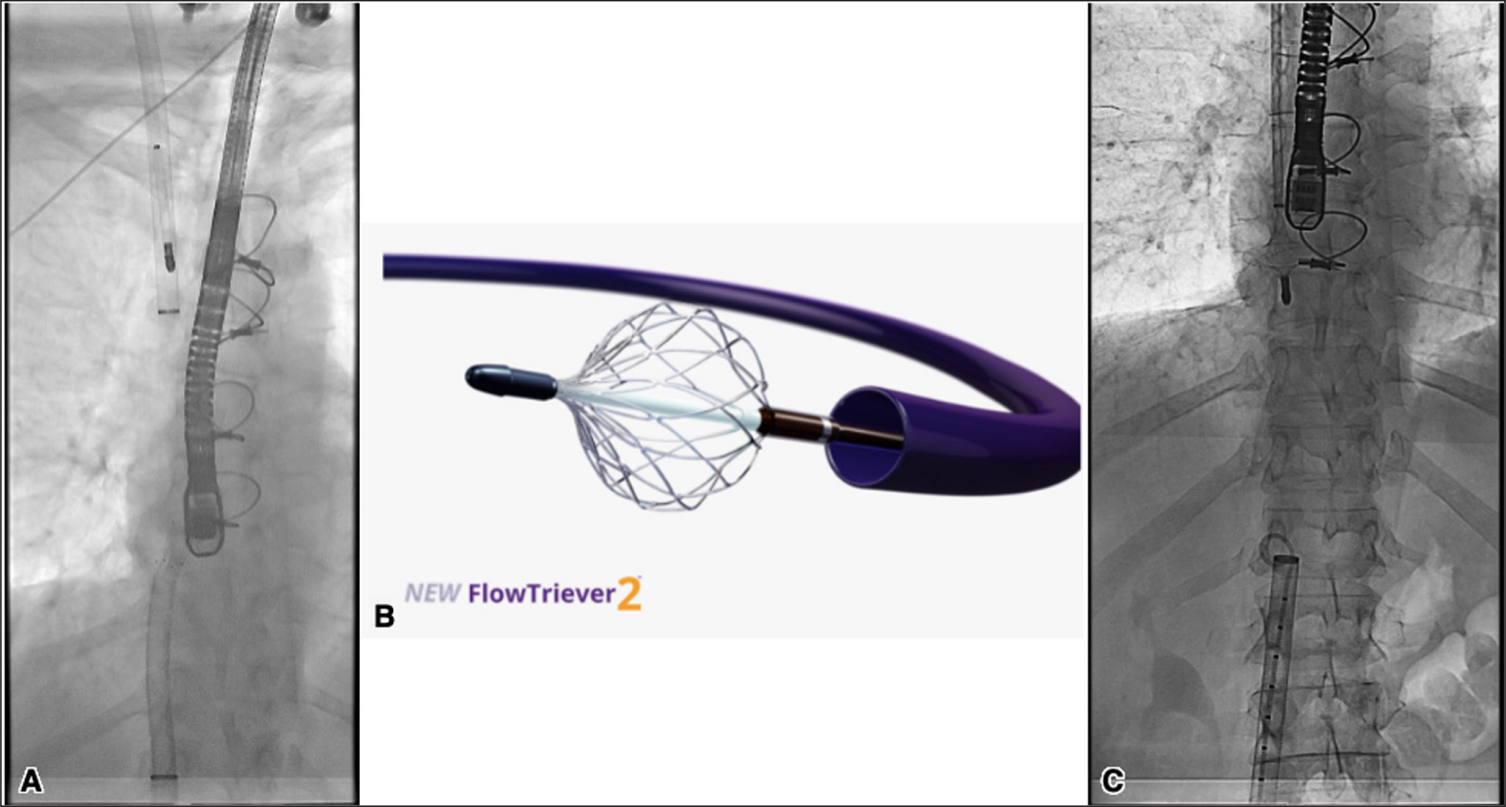
A cine showing the use of the two devices: **(A)** Inari FlowTriever sheath in the right internal jugular vein and the AngioVac from the femoral approach. **(B)** FlowTriever 2 disc. **(C)** FlowTriever catheter’s nitinol mesh disc to prevent clot migration, while the AngioVac catheter is being removed with a mass attached.

First, a veno-venous AngioVac circuit was built in a standard fashion (a 26F Gore DrySeal in the right femoral vein and a 16F return cannula into the left femoral vein), and the mass was engaged with the AngioVac device. However, the mass’s size and chronicity made it impossible to extract it into the AngioVac despite multiple attempts and high-flow suction (Video 1). Next, through the right internal jugular vein, a 24F Inari FlowTriever sheath was advanced to the superior vena cava where the mass originated (Figure 1A).

Next, while the AngioVac catheter was actively catching the mass, the FlowTriever toolkit (the FlowTriever 2 disc, Figure 1B) was used to break up the mass while performing active suction to help push it into the AngioVac cannula (Video 1). In addition, 10 mg tPA was injected onto the mass from the FlowTriever sheath. After multiple attempts to break up the mass, we were able to suction out a significant portion and separate the body from the stalk. However, a big piece of the mass was caught on the tip of the AngioVac, so we again tried to push it into the AngioVac cannula with the FlowTriever 2 disc (Video 1).

To prevent embolization of the detached piece through the right internal jugular (RIJ) FlowTriever sheath, we advanced the FlowTriever catheter (the three nitinol mesh discs) from the RIJ and deployed one disc at the inferior vena cava/right atrial (IVC/RA) junction to prevent migration of the clot upwards to the pulmonary artery while also trying to get the piece into the 26F Gore DrySeal sheath (Figure 1C, Video 1). With the FlowTriever catheter disc open in the IVC/RA junction, the AngioVac catheter was pulled back into the 26F Gore DrySeal sheath while actively suctioning on the mass.

When the AngioVac catheter was pulled out of the body, it contained leftover fragments of the mass and was cleaned out accordingly. The Gore DrySeal sheath was also extracted (exchanged carefully over a wire) and cleaned out. Finally, the sheath was exchanged for a 24F Gore DrySeal. No mass was present in the IVC or anywhere on the right side. TEE showed that the big mass was extracted; however, the peduncle/stalk was still attached to the superior vena cava (SVC) (Video 1).

Through the FlowTriever sheath in the RIJ, a 30 mm Gooseneck snare was placed and navigated to hook around the remaining mass stalk attached to the SVC. Once the AngioVac catheter was engaged in the mass, active suction was applied, and the snare was used to cut the base of the mass, which was successfully extracted through the AngioVac (Video 1). The mass was deconstructed with the Inari device and suctioned with the AngioVac. TEE now showed complete resolution of the mass and its stalk, with no remaining pieces behind (Video 1).

**Figure 2:**
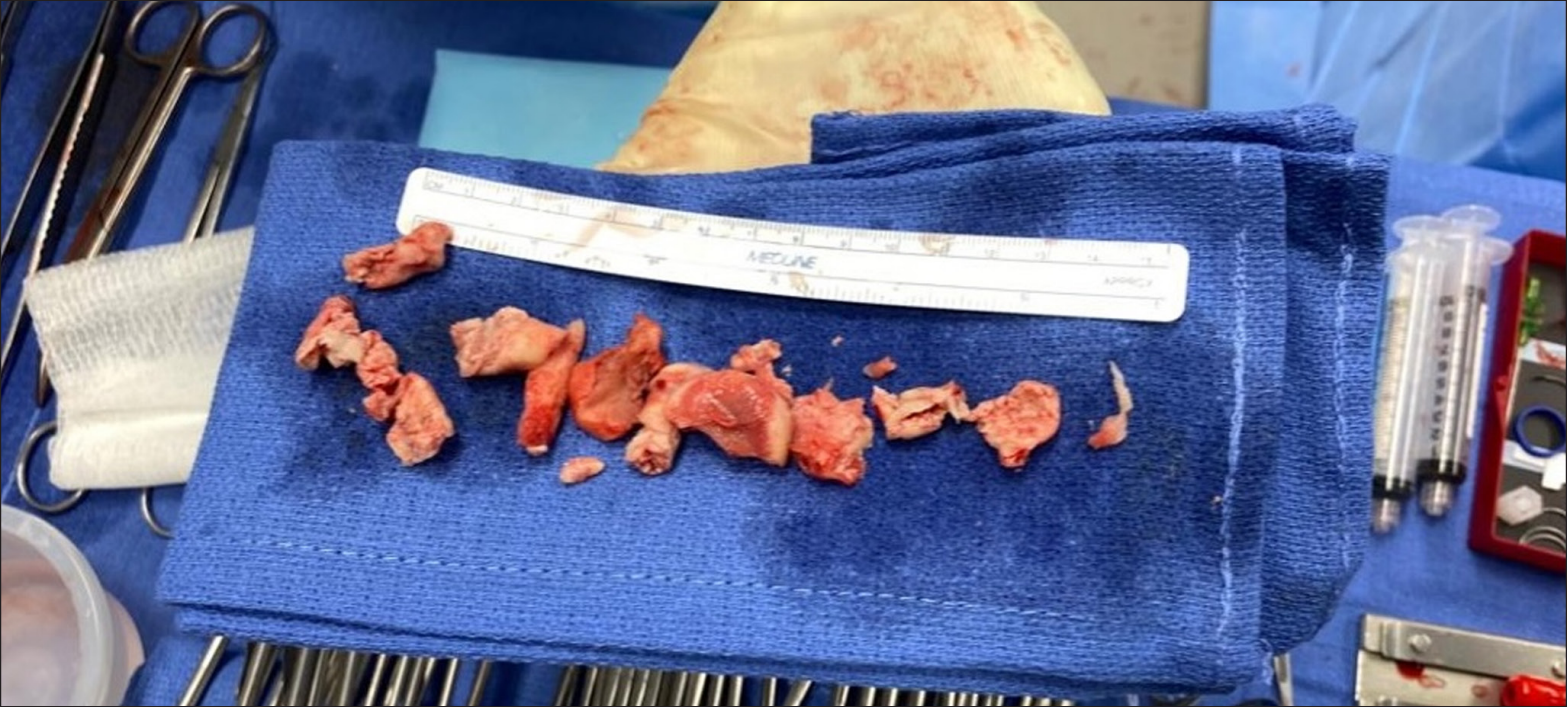
The gross appearance of the mass after successful fragmentation and extraction.

The patient was stable throughout the procedure without any change in vital signs. The final pathology was found to be a thrombus with necrotic and fibrinous tissue. Figure 2 shows the gross appearance of the mass after successful fragmentation and extraction. The patient tolerated the procedure well, with recommendations of surveillance neck and chest CT in 3 months and long-term oral anticoagulation. The patient was discharged home the next day.

## Discussion

This unusual case represents the novel simultaneous use of two large-bore thrombectomy devices to extract a very large mass from the right atrium. It highlights new frontiers in the treatment of complex transcatheter mass extraction. Although this case demonstrated the safety and effectiveness of combining these two technologies, careful planning and execution must be done before performing these procedures in these kinds of cases.

## Conclusion

Concurrent use of the two largest transcatheter thrombectomy devices—hence the names Kong and Godzilla—can provide a powerful addition to the interventional cardiology armamentarium. It offers a realistic option if used carefully and in the right setting. However, the use of these devices is off-label and is not without risks. Their combined use should be considered on a case-by-case basis and weighed against alternatives.
